# Green synthesis of zinc oxide nanoparticles using *Padina pavonica* extract for efficient photocatalytic removal of methylene blue

**DOI:** 10.1038/s41598-024-80757-9

**Published:** 2024-12-31

**Authors:** Ahmed E. Alprol, Ahmed Eleryan, Ahmed Abouelwafa, Ahmed M. Gad, Tarek M. Hamad

**Affiliations:** National Institute of Oceanography and Fisheries, NIOF, Cairo, Egypt

**Keywords:** Green synthesis, Zinc oxide nanoparticles, *Padina pavonica*, Methylene blue, Photocatalysis, Antimicrobial activity, Environmental sciences, Chemistry, Materials science

## Abstract

Dye-laden wastewater poses a significant environmental and health threat. This study investigated the potential of green-synthesized zinc oxide nanoparticles (ZnO NPs), derived from *Padina pavonica* brown algae extract, for the removal of methylene blue (MB) dye. The hypothesis was that utilizing algal extract for ZnO NP synthesis would enhance adsorption capacity and photocatalytic activity for dye removal. The synthesized ZnO NPs, characterized by Infrared Spectroscopy (FTIR), Scanning Electron Microscopy (SEM), Energy Dispersive X-ray Spectroscopy (EDX) and Zeta Potential, demonstrated high adsorption capacity (Qm = 192.308 mg g^−1^) and excellent removal efficiency (> 98%) for MB at low dye concentrations. Langmuir isotherm and pseudo-second-order kinetic models best fit the experimental data, suggesting monolayer adsorption and chemisorption as the primary mechanisms. Notably, the green ZnO NPs exhibited greater photocatalytic activity under direct sunlight irradiation compared to other light sources. Additionally, these nanoparticles displayed antimicrobial properties against various bacteria, indicating potential for water disinfection. This research offers a sustainable and environmentally friendly approach for wastewater treatment utilizing green ZnO NPs for efficient dye removal and potential water disinfection applications.

## Introduction

Water pollution is a major global concern, with the textile industry being a significant contributor due to the release of large volumes of dye-laden wastewater. Dyes are complex organic compounds that impart color to textiles and other materials^1^. Many of these dyes are toxic, carcinogenic, and mutagenic, posing serious threats to aquatic ecosystems and human health^2^. Methylene blue (MB), a cationic thiazine dye, is widely used in various industries and is known to cause harmful effects on aquatic organisms and humans, including respiratory distress, nausea, vomiting, and eye irritation^3,4^. Therefore, it is crucial to develop efficient and sustainable methods for removing dyes from wastewater before their release into the environment. Several conventional methods have been employed for dye removal, including coagulation, flocculation, adsorption, membrane filtration, and biological treatment^5,6^. However, these methods often suffer from limitations such as high cost, low efficiency, generation of secondary pollutants, and complex operation. In recent years, advanced oxidation processes (AOPs) have gained considerable attention for their ability to degrade organic pollutants effectively^7^. With its capacity to use solar energy to produce reactive oxygen species (ROS) that can break down organic pollutants, photocatalysis has become one of the most promising AOPs for dye removal^8,9^. Additionally, photocatalysis is a highly advanced oxidative process that, like adsorption, swiftly and non-selectively oxidized organic contaminants. It is also an inexpensive and easy way to get rid of anionic and cationic dyes. The creation of materials with multiple applications is far more beneficial than the creation of materials with specialized uses. Among other nano metal oxides, zinc oxide and its composites were used as an adsorbent and photocatalyst to remove a variety of dyes. Zinc oxide and its composites were used as an adsorbent and photocatalyst for the removal of many dyes among other nano metal oxides^10^.

Zinc oxide (ZnO), a wide bandgap semiconductor, has emerged as a promising material for various applications, particularly in the field of environmental remediation. ZnO’s exceptional properties, including its high photocatalytic activity, biocompatibility, and low cost, have positioned it as a leading material for advanced oxidation processes (AOPs), a key approach in tackling organic pollutant degradation^11^. However, the conventional synthesis methods for ZnO nanoparticles often involve the use of harsh chemicals and high energy consumption, making them environmentally undesirable. While the applications of ZnO in environmental remediation hold tremendous promise, certain challenges and disadvantages warrant consideration: while ZnO’s wide bandgap (3.37 eV) limits its photocatalytic activity to UV light, which comprises only a small fraction of the solar spectrum. This limitation reduces the efficiency of ZnO photocatalysts under natural sunlight conditions. The tendency for ZnO nanoparticles to agglomerate in solution can reduce their surface area and therefore decrease their effectiveness^12^. Agglomeration limits the contact between the photocatalyst and pollutants, diminishing its performance. Recovery and reuse of ZnO nanoparticles following treatment can pose challenges. Ensuring the complete removal of ZnO nanoparticles from treated wastewater is crucial to avoid potential toxicity and minimize secondary pollution.

Zinc oxide is a widely used photocatalyst due to its high photocatalytic activity, low cost, and environmental friendliness^13^. However, the conventional synthesis methods of ZnO nanoparticles often involve the use of hazardous chemicals and high energy consumption. Therefore, green synthesis methods using algae extracts have been explored as a sustainable alternative^14^. Green synthesis methods often utilize algae extracts or other biological materials, leading to the presence of residual organic compounds on the nanoparticle surface. These compounds can act as capping agents, influencing particle size, morphology, and stability^15^. The use of green synthesized nanoparticles for dye removal is a growing area of research due to its environmental benefits. Several studies have explored the use of ZnO NPs for dye adsorption, demonstrating their effectiveness in removing various types of dyes. The efficiency of dye removal depends on several factors, including the size, morphology, and surface properties of the nanoparticles, as well as the chemical structure of the dye molecules^16^. Algal extracts contain various phytochemicals that can act as reducing and capping agents for the synthesis of nanoparticles. This approach offers several advantages, including being environmentally friendly, cost-effective, and producing stable nanoparticles with enhanced properties^17^. *Padina pavonica* brown algae are a rich source of bioactive compounds such as polysaccharides, polyphenols, and flavonoids^18^. These compounds have the potential to act as reducing and stabilizing agents for the synthesis of ZnO NPs. Moreover, the presence of these bioactive compounds may enhance the photocatalytic activity of ZnO NPs by increasing their surface area and reducing the recombination rate of photogenerated electron-hole pairs^19^. ZnO nanoparticles have multiple functions^20^. Firstly, they act as an adsorbent due to the presence of a single pair of electrons on the oxygen atom, which promotes the adsorption of a π electron in the conjugated system of MB by forming a (n-π) bond. Secondly, they act as a photocatalyst that can break down organic materials. Lastly, they function as an electrode material for the electrochemical degradation of positively charged dyes by gaining a negative charge upon electric current passage. Also, Zinc oxide (ZnO) is a versatile inorganic compound known for its broad range of applications, particularly in the medical and healthcare industries due to its antimicrobial properties. In recent years, ZnO has gained significant attention for its efficacy as an antibacterial agent. This efficacy is primarily attributed to its unique physicochemical properties, including its ability to generate reactive oxygen species (ROS), release zinc ions, and interact with bacterial cell membranes, leading to the disruption of cellular processes^11^.

Therefore, the present study aims to evaluate the potential of green synthesized ZnO nanoparticles as a sustainable and efficient adsorbent for dye removal from wastewater. Characterize the synthesized ZnO NPs using various techniques. Investigate the photocatalytic activity of ZnO NPs for the removal of MB dye from aqueous solutions under direct sunlight irradiation. Optimize the operational parameters affecting the photocatalytic degradation process. And analyze the adsorption kinetics and isotherms to understand the underlying mechanisms. Finally, the antimicrobial properties of ZnO nanoparticles were evaluate against some microbial in gram positive and negative bacteria.

## Materials and methods

### Reagents and materials

**Fig. 1:**
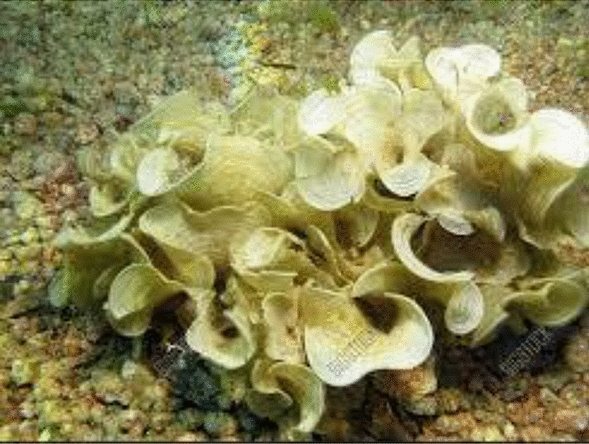
*Padina pavonica* as Brown Algae, taken from Shutterstock^21^.

*Padina pavonica* brown algae (Fig. 1) were collected from the Ras Sidr area on the Red Sea coast, Egypt. Zinc nitrate hexahydrate Zn (NO_3_)_2_· 6H_2_O was purchased from sigma Aldrich with purity (99.9%). Methylene blue dye was purchased from novia Hexachem (≥ 99.0%). Sodium hydroxide NaOH (98%) and hydrochloric acid HCl (37%) were used for pH adjustment. All chemicals used were of analytical grade and used without further purification.

### Green synthesis of Zno nanoparticles using *Padina pavonica* brown algae extract

Green synthesis methods, employing biological materials like algal extracts, offer an environmentally friendly alternative to conventional chemical approaches. This method is attractive because it often avoids the use of toxic chemicals and harsh conditions, making it a sustainable route for nanoparticle production^22,23^.

#### Algae preparation

*Padina pavonica* as brown algae, a marine algae species known for its rich bioactive compounds, was chosen as the source for the synthesis. The algae were thoroughly washed with distilled water to remove any adhering sand, debris, or other impurities. This ensures the purity of the extract and prevents contamination of the nanoparticles. The washed algae were dried in an oven at 60 °C for 24 h. This temperature is sufficient to remove moisture without degrading the bioactive compounds within the algae. The dried algae were ground into a fine powder using a mixer. This step increases the surface area of the algae, facilitating the extraction process^24^.

#### Algae extract preparation

10 g of the algae powder were added to 100 mL of distilled water and boiled for 30 min. This process extracts the bioactive compounds from the algae. The boiling time of 30 min is chosen to ensure efficient extraction while minimizing the degradation of bioactive compounds. The extract was filtered through Whatman No. 1 filter paper. This removes any undissolved algae particles, leaving behind a clear extract. The filtrate was stored at 10 °C. This temperature helps to preserve the bioactive compounds and prevent microbial growth^25^.

#### ZnO nanoparticle synthesis

Co-precipitation method was used since 50 mL of the algae extract was added to 50 mL of 0.1 M zinc nitrate hexahydrate Zn (NO_3_)_2_.6H_2_O solution under constant stirring. This initiates the reaction between the bioactive compounds in the extract and the zinc ions. The pH of the reaction mixture was adjusted to 10 using 0.1 M NaOH solution. The mixture was stirred at room temperature (approximately 25 °C) for 2 h to allow for complete precipitation. The resulting precipitate, containing the ZnO nanoparticles precursor, was collected by centrifugation at 5000 rpm for 10 min. The precipitate was then washed several times with distilled water to remove any residual impurities from the extract. The washed precipitate was then dried in an oven at 80 °C for 12 h to remove residual moisture. Finally, the dried precipitate was subjected to calcination at 300 °C for 2 h. This high-temperature treatment resulted in the conversion of the ZnO precursor into the desired ZnO nanoparticles^26^.

### Characterization of green synthetized ZnO NPs

The physicochemical properties of the green synthetized ZnO NPs that is formed will be described using the following techniques: To find distinctive peaks connected to the functional groups found in the nanocomposite, FTIR spectroscopy was utilized to scan the sample over the desired spectral range, which is normally between 4000 and 400 cm^−1^ (Bruker -Version 7.2). Zeta Potential Report was used to measure zeta potential in line with this, at 25.0 °C. The experiment consisted of 20 zeta runs at a count rate of 34.6 kcps. At a distance of 2.00 mm, measurements were taken inside a clear, disposable zeta cell. However, green ZnO NPs material with distinguishing characteristics can be recognized by Energy Dispersive X-ray spectroscopy (EDX) study utilizing the Japanese JEOL JSM-IT200 apparatus and Scanning Electron Microscopy (SEM) analysis. Using X-ray diffraction (XRD, Bruker D & Advance, Germany) with CuKα as the radiation source (40 kV, step size 0.02, scan rate 0.5 min^−1^, 20° ≤ 2θ ≤ 80°), the crystal phases of the produced powders were identified.

### Photocatalytic experiments

The photocatalytic activity of synthesized ZnO was evaluated by the photodegradation of methylene blue under direct solar radiation, where the average daily temperature was 24 ± 2 °C. The effects of various operational parameters, including initial dye concentration (20–100 ppm), contact time (5–180 min), adsorbent dosage (10–50 mg), and pH (3–12), were studied for the degradation of MB. In a typical experiment, a known amount of ZnO nanoparticles was added to a 100 mL solution of MB dye at a specific concentration and pH. The mixture was not agitated for a predetermined contact time. After degradation, the mixture was centrifuged to remove the catalyst, and the residual dye concentration in the supernatant was determined using a UV–Vis spectrophotometer at a wavelength of 665 nm. Then, the absorption was converted to the concentration through the standard calibration curve.

The adsorption capacity (*q*_*e*_) and removal efficiency (%) were calculated using the following equations. ^4^^,^^27^:

1 $$q_{e} = \, \left( {C_{o} - \, C_{e} } \right) \, \times {\text{ V }}/{\text{ M}}$$

2 $${\text{Re}} moval \, efficiency \, \left( \% \right) \, = \, \left( {C_{o} - \, C_{e} } \right) \, \times \, 100 \, /C_{o}$$

where: *C*_*o*_ is the initial dye concentration (ppm), *C*_*e*_ is the equilibrium dye concentration (ppm), V is the volume of the solution (L) and M is the mass of the adsorbent (g).

### Adsorption isotherms

The adsorption isotherms were studied by fitting the data to Langmuir, Freundlich, and Tempkin isotherm equations. For the equilibrium investigations, the Langmuir, Freundlich, and Tempkin isotherm (Eqs. (3), (4), (5), respectively) were employed. By building the appropriate model, the distinct environmental conditions of each sorption system can be represented. The sorption capacity residual concentration and the adsorbate fixed temperature are determined by the adsorption isotherm.

3 $$q_{e} = \frac{{Q_{m} K_{a} C_{e} }}{{1 + K_{a} C_{e} }}$$

4 $$q_{e} = K_{F} C_{e}^{1/n}$$

5 $$q_{e} = B \, \ln \, A + \, B \, \ln C_{e}$$

where: *Q*_*m*_ is the solute’s highest adsorption capacity (mg g^─1^) and *K*_*a*_ is the sorption equilibrium constant (L mg^─1^), which is connected to Langmuir’s apparent energy of adsorption. *K*_*F*_ is the Freundlich constant revealing of the comparative sorption capacity of the adsorbent material correlated to the bonding energy. A (L g^─1^) is Tempkin isotherm constant also, called equilibrium binding constant.

### Adsorption kinetics

The sorption kinetics of MB on Zinc Oxide Nanoparticle were analyzed by fitting the experimental data to pseudo-first-order, pseudo-second-order, Elovich, Film Diffusion and intraparticle diffusion models^28^.

#### Kinetic model of pseudo-first-order

The following equation represents the linear form of the generalized pseudo-first-order equation:

6 $$Log \, \left( {q_{e} - \, q_{t} } \right) \, = \, \log q_{e} - \, k_{1} t \, /2.303$$

where: *Q*_*e*_ stands for the amount of dyes adsorbed at equilibrium (mg g^─1^), *q*_*t*_ for the amount of dyes adsorbed at time t (mg g^─1^), and *K*_*1*_ for the pseudo-first-order rate constant (min^─1^). Plotting log (*q*_*e*_-*q*_*t*_) versus (t) should yield a linear connection between *k*_*1*_ and *q*_*e*_, which can be evaluated using the slope and intercept.

#### Pseudo-second order kinetic model

The pseudo-second-order equation was written as follows:

7 $$t/q_{t} = 1/K_{2} q_{e}^{2} + t/q_{e}$$

Plotting (*t/q*_*t*_) versus (*t*) yields a linear connection, and the slope and intercept may be used to compute the values of the *q*_*e*_ and *K*_*2*_ parameters, respectively. Where *K*_*2*_ denotes the second-order rate constant (g mg^─1^ min^─1^).

#### The intraparticle diffusion model

The intraparticle diffusion equation is explored as follows:

8 $$q_{t} = K_{dif} t^{1/2} + \, C$$

where: *q*_*t*_ (mg g^─1^), is the quantities of dye adsorbed at time *t*. Also, intercept denotes the value C when the adsorption mechanism follows the intraparticle diffusion process. The values of the intercept provide an idea about the thickness of boundary layer; i.e., the larger the intercept, the greater the boundary layer effect and *K*_*dif*_ (mg g^─1^ min^−0.5^) is the intraparticle diffusion rate constant, which is calculated using the regression line’s slope.

### Evaluation of antibacterial activity

The antibacterial activities of green synthesized ZnO NPs derived from *Padina* brown algae were assessed using the well diffusion method on Mueller–Hinton agar (MHA). Inhibition zones were measured in millimeters (mm). Bacterial Strains: *Staphylococcus aureus* (ATCC 25923), *Escherichia coli* (ATCC 8731), *Pseudomonas aeruginosa* (ATCC 9027), *Vibrio damsella, Klebsiella pneumoniae* (ATCC 13883), *Enterococcus faecalis* (ATCC 29212) served as reference strains for the antibacterial assay of green ZnO NPs. In brief, MHA agar plates were inoculated with the bacterial strains under aseptic conditions. Wells with a diameter of 6 mm were filled with 50 μl of the test samples and incubated at 37 °C for 24 h. After incubation, the diameters of the growth inhibition zones were measured. Single colonies (18 to 24 h old) from agar plates were used to prepare bacterial suspensions with a turbidity of 0.5 McFarland (equivalent to 1.5 × 10^8^ CFU/ml). The turbidity of the bacterial suspensions was measured at 600 nm. All tests were performed in triplicate^29^.

## Results and discussion

### *Padina pavonica*-mediated green creation of ZnO NPs

The aqueous extract of algae contains carbohydrates, fats, vitamins, fatty acids, bioactive metabolites like pigments and polyphenols, (phycobilin, carotenoids, and chlorophyll) that function as stabilizing and reducing agents for the formation of a wide range of metal and metal oxide nanoparticles^30^. In the current study, the *Padina pavonica* extract contains a diverse mix of biomolecules, including polysaccharides and phenolic compounds, that play crucial roles as reducing agents and capping agents in the synthesis of ZnO NPs. The active metabolites involved in the aqueous extract of *Padina pavonica* extract contains biomolecules acting as reducing agents and catalyzes Zinc precursor salt reduction of zinc nitrate to zinc oxide nanoparticles. The creation of a white hue in the present investigation upon blending the algal aqueous extract with Zn(NO_3_)_2_·6H_2_O metal precursor signifies the accomplishment of ZnO NPs production. The fabrication of ZnO NPs using an aqueous extract of *Padina pavonica* brown algae and zinc nitrate hexahydrate solution involves a series of steps that leverage the reducing and stabilizing properties of bioactive compounds present in the algae. One of two possible mechanisms could be responsible for the production of ZnO NPs from an algal aqueous extract: the first is the ability of biomolecules and active metabolites present in the aqueous extract to chelate Zn^2+^ to form a complex of Zn(OH) (Complexation and Precipitation(, which is then collected and calcined at 300 °C for two hours to form a white powder that is represented as ZnO NPs in Eqs. (9), (10), ^31^.

9 $$\begin{array}{*{20}c} {Zn\left( {NO_{3} } \right)_{2} \cdot 6H_{2} O + Padina\,aqueous\,extract \to \left[ {Zn\left( {Biomolecule} \right)_{2} } \right]^{2 + } + 2NO_{3}^{ - } + 6H_{2} O} \\ {\left[ {Zn\left( {Biomolecule} \right)_{2} } \right]^{2 + } + 2OH^{ - } \to Zn\left( {OH} \right)_{2} \downarrow + 2Biomolecule)} \\ \end{array}$$

At pH 10, the hydroxide ions (OH^−^) from NaOH react with zinc ions (Zn^2+^) to form zinc hydroxide Zn(OH)_2_, which is a white precipitate. The washed precipitate is calcinated at a temperature of 300 °C for 2 h. Upon calcination at 300 °C, the Zn(OH)_2_ is converted into ZnO nanoparticles^32^ as showed in Eq. (10):

10 $$Zn\left( {OH} \right)_{2} \mathop{\longrightarrow}\limits^{300^\circ C}ZnO+ \, H_{2} O\left( g \right)$$

The Zn^2+^ ions complex with biomolecules from the extract. The complex then reacts with hydroxide ions (OH^−^) from the extract or water to form a precipitate of zinc hydroxide. Similarly, the reducing agents in the extract reduce Zn^2+^ to metallic zinc as the following Eq. (11). Zn^0^ then reacts with dissolved oxygen to form ZnO nuclei. Algal metabolites cap the ZnO nuclei, preventing aggregation and stabilizing the nanoparticles. Correspondingly, the second pathway might involve the active metabolites in the aqueous extract reduce Zn^2+^ to create zinc metal (Zn^0^). The ZnO nuclei are then produced through a reaction with dissolved oxygen. The freshly generated ZnO is then capped by a few algae metabolites, which may improve the nanomaterials’ stability and stop them from aggregating.

11 $$Zn\left( {NO_{3} } \right)_{2} \cdot6H_{2} O \, + Padina\;aqueous \, extract \to Zn \downarrow \, + \, 2NO_{3}^{ - } \, + \, 6H_{2} O \, + \, Other \, products$$

## Characterization of green synthetized ZnO NPs

### Analysis of zeta potential results

**Fig. 2:**
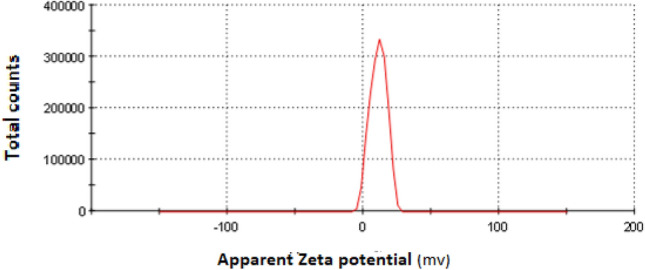
Zeta potential measurement of green synthetized ZnO NPs in aqueous solution.

Zeta potential is a valuable tool for characterizing the surface charge of nanoparticles. It provides information about the stability of colloidal suspensions and the potential for interaction with other charged species, such as dyes or pollutants. Based on the provided zeta potential in Fig. 2. Green synthetized ZnO NPs exhibit a positive zeta potential of 11.0 mV. A positive zeta potential signifies that the surface of the nanoparticles carries a net positive charge. Methylene blue (MB) is a cationic dye, meaning it possesses a positive charge. In this case, the positive zeta potential of the Green synthetized ZnO NPs suggests an electrostatic repulsion between the nanoparticle surface and the MB dye molecules. This repulsion could hinder the adsorption of MB dye onto the nanoparticles. This can occur due to the presence of positively charged functional groups on the nanoparticle surface or the preferential adsorption of cations from the surrounding solution^33^. While the positive zeta potential indicates unfavorable electrostatic interactions for MB dye adsorption, other factors could still influence the adsorption process. Even with electrostatic repulsion, other adsorption mechanisms, such as van der Waals forces, hydrogen bonding, or dipole–dipole interactions, might contribute to MB dye uptake^33^. The zeta potential and surface charge of nanoparticles can vary with pH. Investigating the effect of pH on MB dye adsorption could reveal optimal conditions for the process.

### Analysis of Fourier-transform infrared spectroscopy (FTIR)

**Fig. 3:**
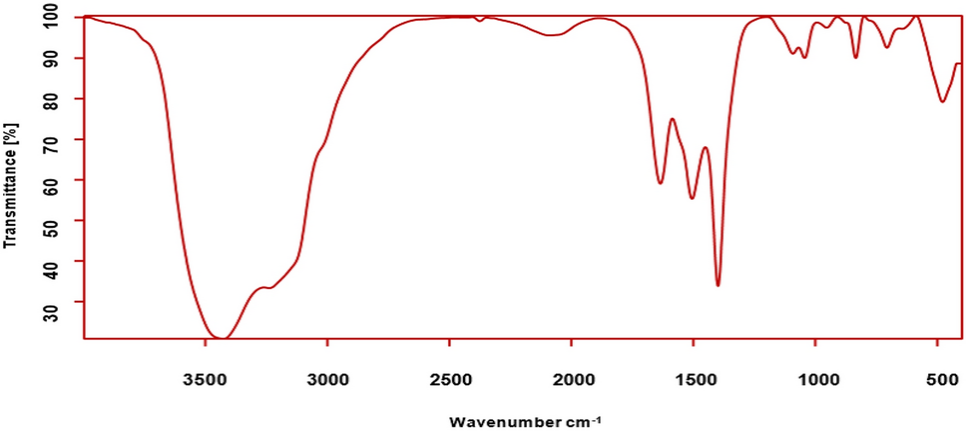
FTIR spectroscopy for green synthetized ZnO NPs from *Padina* algae.

FTIR spectroscopy is a powerful and versatile tool used to determine the functional groups present in the sample, as well as its chemical structure and composition^34^. The provided results in Fig. 3 and Table 1 shows the results of FTIR spectroscopy for a sample containing green synthetized ZnO NPs from *Padina* algae besides possible functional groups and its role in adsorption process. The spectrum range between 500–4000 (cm^−1^). As discussed in Table 1, the FTIR spectrum suggests the presence of various functional groups, including: O–H and NH_2_ groups in range 500–4000 (cm^−1^) these could originate from the surface of the ZnO NPs, residual water molecules, or functional groups within the algae-derived organic material. While, C = C and C-H groups are likely associated with the organic components of the nanocomposite derived from the *Padina* algae. Besides, C–O groups could also originate from the algae-derived organic material or from interactions between the ZnO nanoparticles and organic molecules. Also, Peaks in the fingerprint region, particularly around 706 cm^−1^ and 478 cm^−1^, support the presence of metal–oxygen bonds (Zn–O)^16^.

**Table 1:** Referring spectral and literature relevant of FTIR to green synthetized ZnO NPs from algae.

Wavenumber (cm^−1^)	Possible functional group	Role in adsorption process
3426.749	O–H stretching (alcohols, phenols, or adsorbed water)	Potential hydrogen bonding interaction with MB dye
2374.944	C≡N or C≡C stretching	
2090.107	C≡C stretching (possibly allenes or other cumulative double bond systems)	
1634.860	C = O stretching (carbonyl groups)	Potential interaction with MB dye through dipole–dipole interactions or hydrogen bonding
1505.233	Aromatic C = C stretching	Could contribute to π-π interactions with the aromatic rings of MB dye
1398.846	C–H bending	May indirectly influence adsorption by affecting the overall molecular conformation
1091.980	C-O stretching (alcohols, ethers, or carbohydrates)	Potential hydrogen bonding interaction with MB dye
1043.947		
953.286	= C–H bending (alkenes)	May indirectly influence adsorption by affecting the overall molecular conformation
834.245	C–H bending (aromatics)	
706.304		
478.645	Metal–oxygen (Zn–O) stretching	Confirms the presence of zinc oxide in the nanocomposite

### Scanning *electron* microscopy (SEM)

**Fig. 4:**
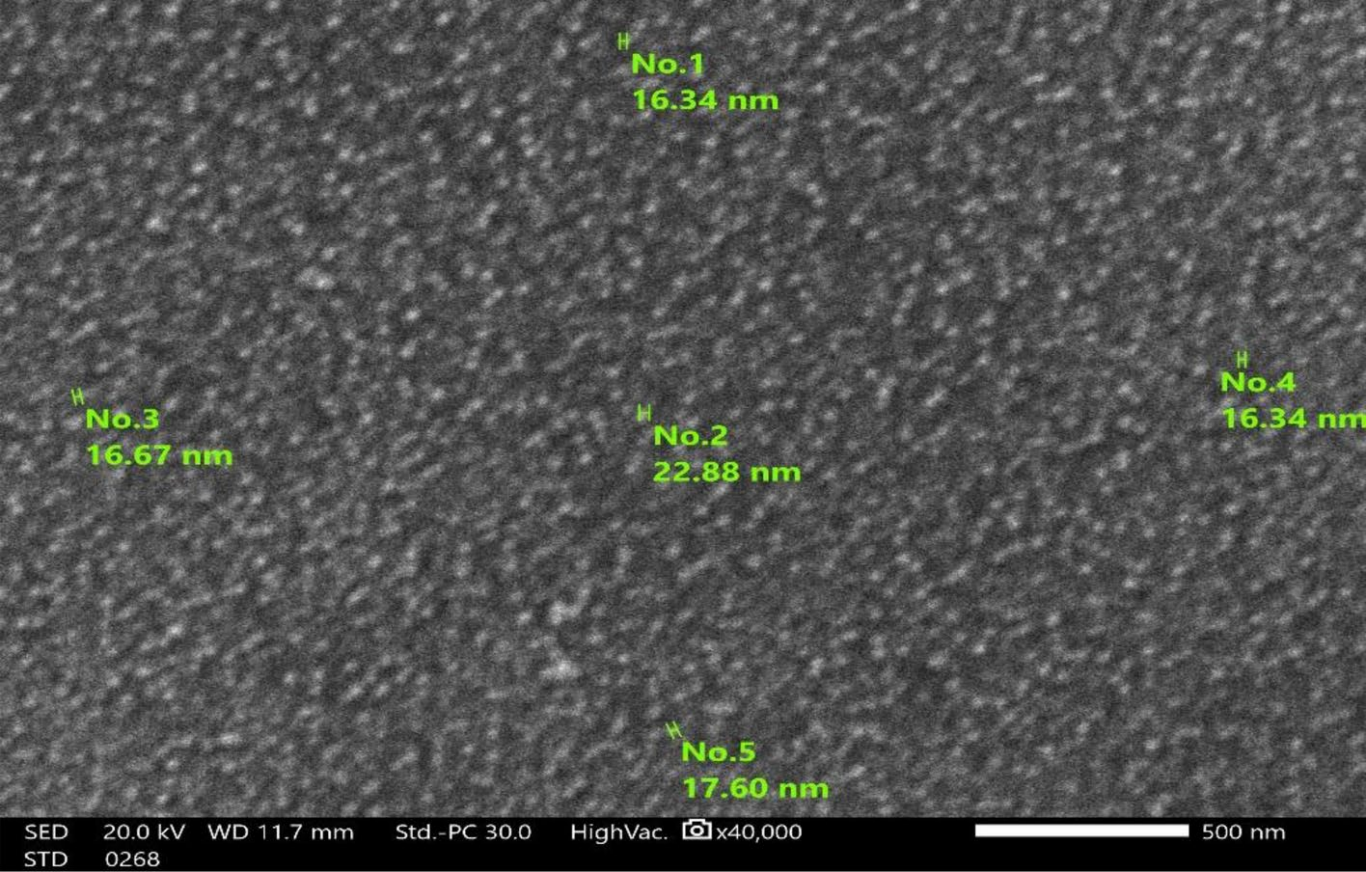
SEM green synthetized ZnO NPs.

SEM provides valuable information about morphology and size of green synthetized ZnO NPs. The image of SEM was captured at a magnification of 40,000× , allowing for clear visualization of individual nanoparticles and their surface features. The Green synthetized ZnO NPs exhibit a predominantly spherical morphology. Figure 4 reveals a diverse landscape of particles with some degree of agglomeration present. The surface texture appears relatively smooth, with minimal visible porosity. Based on the measurements of SEM, the particle size ranges from approximately 16.34 nm to 22.88 nm. This suggests a relatively narrow size distribution, although a more comprehensive analysis of multiple images or particle size distribution software would provide a more accurate assessment. The SEM analysis confirms the successful synthesis of ZnO NPs with a predominantly spherical shape and a size range within the nanoscale^35^. The observed agglomeration is a common phenomenon in nanoparticle synthesis and can influence the material’s properties.

### Energy-dispersive X-ray spectroscopy (EDX) analysis

**Fig. 5:**
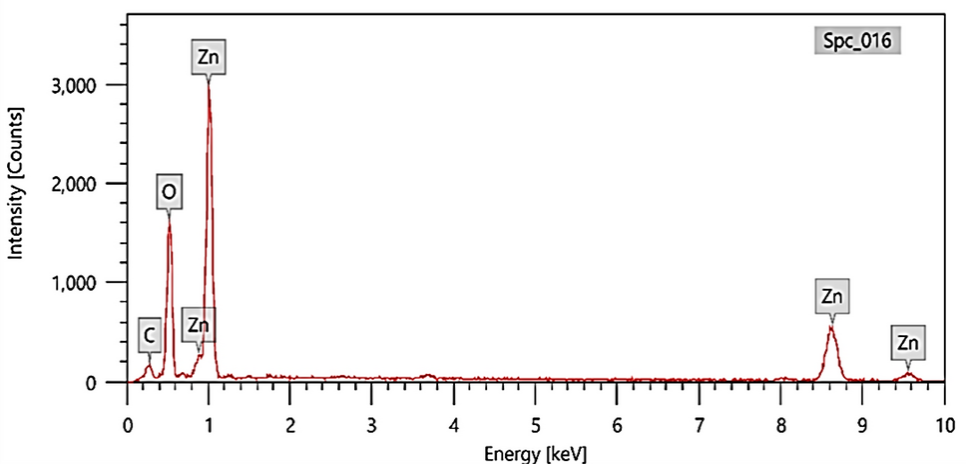
EDX analysis of Green synthetized ZnO NPs by *Padina* algae.

To confirm the presence of Zinc and Oxygen in the sample, and thus prove the composition of Green synthetized ZnO NPs, Energy-Dispersive X-ray Spectroscopy (EDX) analysis is crucial^36^. The provided EDX data in Fig. 5 and Table 2 reveals valuable insights into the elemental composition of your green synthesized ZnO NPs. It showed that the presence of carbon (10.52 wt%, 21.78%) is significant and likely originates from the organic precursors or capping agents used in the green synthesis process. These organic components could be responsible for stabilizing the nanoparticles and preventing agglomeration. Also, oxygen is a major component (37.63 wt%, 58.49%) due to the formation of zinc oxide. In addition, Zinc constitutes a substantial portion (51.85 wt%, 19.73%) confirming the presence of ZnO in the sample. The data does not indicate the presence of any impurities or unexpected elements, suggesting a relatively pure ZnO NPs sample. The ratio of Zn to O is not exactly 1:1 as expected in pure ZnO, confirming the formation of Zinc Oxide. The relative intensities of these peaks would provide insights into the stoichiometry of the compound. This could be due to surface defects, oxygen vacancies, or the presence of organic residues on the nanoparticle surface. Likewise, the significant carbon content suggests the presence of organic molecules on the surface of the ZnO NPs. These molecules could influence the surface properties such as hydrophilicity, surface charge, and interaction with other molecules like MB dye. Understanding the surface properties is crucial as they play a key role in the adsorption process of dyes from aqueous solutions. Carbohydrates, proteins, or polysaccharides from an algal extract may act as capping agents and explain the presence of C^37^. The presence of carbon suggests potential functional groups (e.g., carboxyl, hydroxyl) which can interact with dye molecules through electrostatic interactions, hydrogen bonding, or other mechanisms, facilitating adsorption.

**Table 2:** Element analysis of green synthetized ZnO NPs by *Padina* algae.

Element	Mass %	Atom %
C	10.52 ± 0.21	21.78 ± 0.43
O	37.63 ± 0.36	58.49 ± 0.55
Zn	51.85 ± 0.75	19.73 ± 0.29
Total	100.00	100.00

### X-ray diffraction (XRD)

**Fig. 6:**
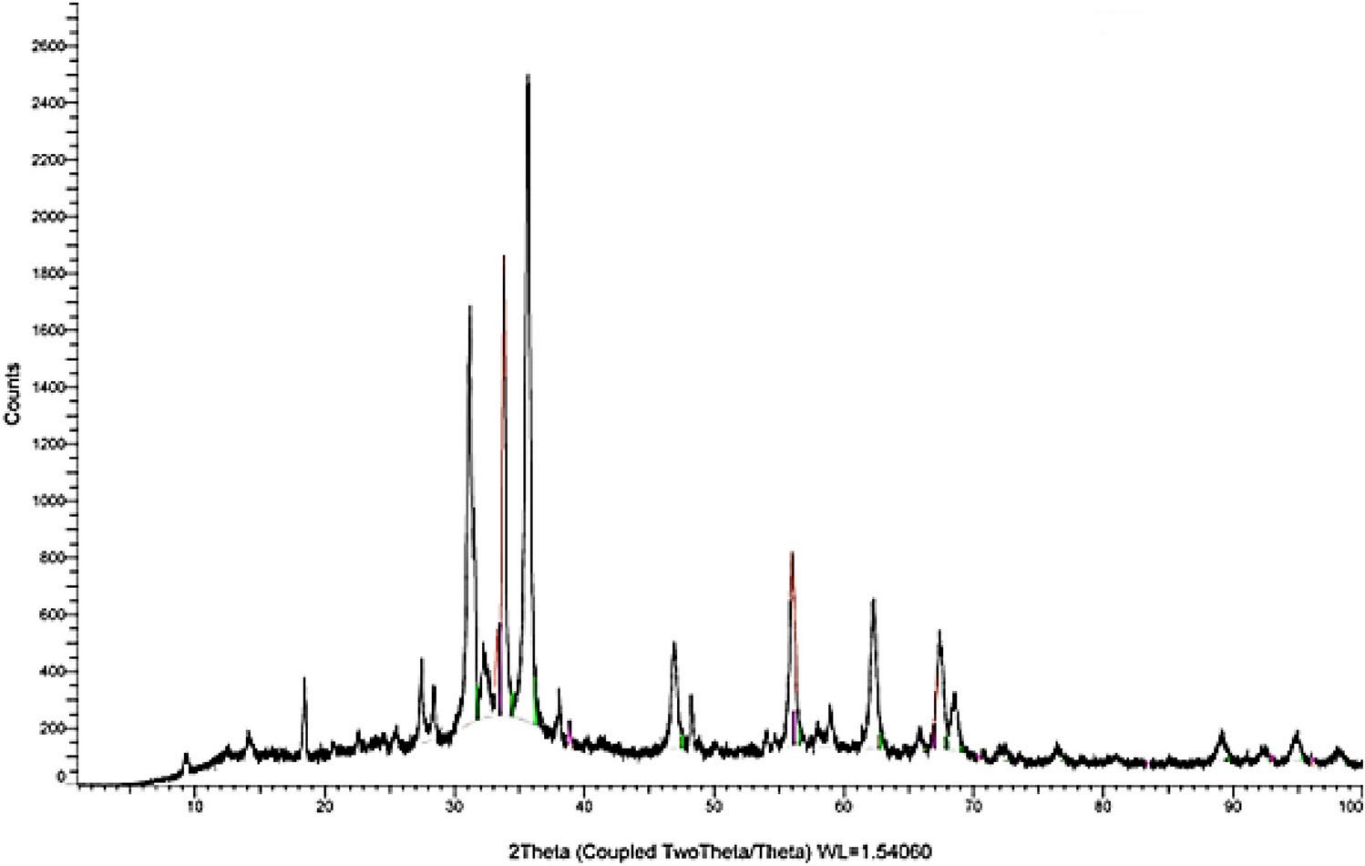
The XRD pattern of the synthesized ZnO nanoparticles.

XRD analysis is a fundamental technique used to investigate the crystalline structure, phase composition, and average particle size of zinc oxide (ZnO) nanoparticles synthesized using *Padina pavonica* brown algae extract. This method provides essential insights into the material’s structural properties, which are vital for various applications in fields such as electronics, optics, and catalysis. The XRD pattern of the synthesized ZnO nanoparticles, as depicted in Fig. 6 reveals distinct diffraction peaks characteristic of a hexagonal wurtzite structure. The observed peaks appear at specific Bragg angles (2θ) of 31.7°, 34.4°, 36.2°, 47.5°, 56.5°, 62.8°, and 67.7°. These peaks correspond to the (100), (002), (101), (102), (110), (103), and (112) crystallographic planes, as indexed by Miller indices (hkl). The hexagonal wurtzite structure is known for its favorable properties, including high thermal and chemical stability.

The positions of the peaks align well with the standard reference patterns provided by the Joint Committee on Powder Diffraction Standards (JCPDS), specifically reference file no. 89–1397. The matching of the experimental peaks with the JCPDS standards confirms the purity and crystalline quality of the ZnO nanoparticles, indicating that no significant impurities were present during synthesis. This purity is crucial for ensuring the desired properties of the nanoparticles in their applications.

These data again give the information about the formation of hexagonal wurtzite structure for all ZnO nanoparticles. The average size of ZnO NPs was calculated from the highest intense peak (101) using Debye–Scherrer equation as below.

12 $$D \, = \, k\lambda /{\ss}\cos \theta$$

where: d is particle size of the crystal, k is Scherer’s constant (0.94), λ is X-Ray wavelength (0.15406 nm), ß is the width of the XRD peak at half height and θ is the Bragg diffraction angle. β = 5 (full width at half maximum in radians), θ = 36.2^∘^ (Bragg angle in degrees). The crystallite size D is approximately 35.89 nm. Substituting these values into the equation yields an average crystallite size of approximately 35.89 nm. This small crystallite size is indicative of a high surface area-to-volume ratio, which can enhance the reactivity and performance of the nanoparticles in various applications, including photocatalysis and antimicrobial activity^38^.

### UV–Vis absorption spectra of methylene blue dye

**Fig. 7:**
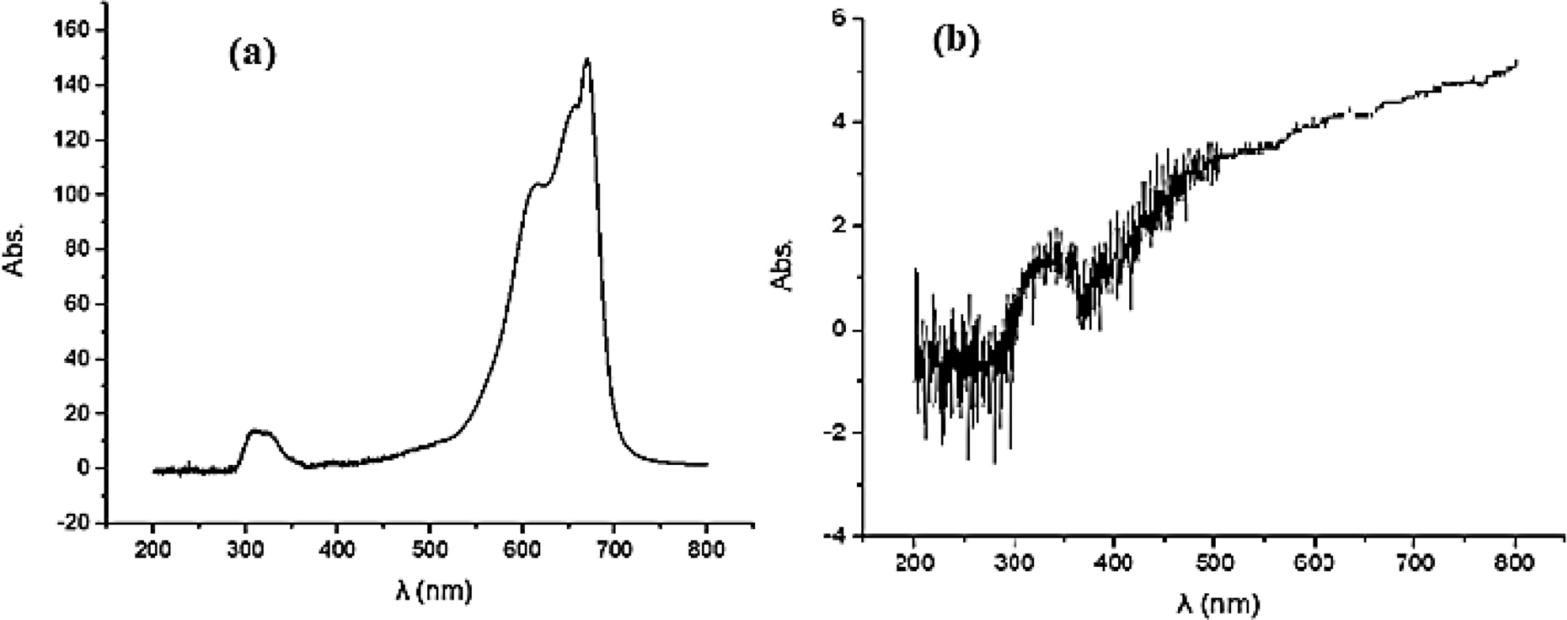
UV–Vis Absorption Spectra of Methylene Blue Dye Before and After Photocatalytic Treatment with Green-Synthesized ZnO Nanoparticles.

UV–Vis spectra analysis of methylene blue (MB) dye before and after treatment with green-synthesized ZnO nanoparticles was presented in Fig. 7. UV–Vis spectrum in Fig. 7a shows the absorbance of MB dye in its aqueous solution before the addition of ZnO nanoparticles. The prominent absorption peak around 660–680 nm is characteristic of MB dye and reflects its high concentration in the solution^39^. There is also a smaller peak around 300 nm, likely due to the π–π* transition associated with the aromatic rings in the dye molecules. While, Fig. 7b spectrum illustrates the absorbance of MB dye after being treated with ZnO nanoparticles under sunlight. The absorbance at 660–680 nm is significantly reduced, indicating a substantial decrease in dye concentration. This change is attributed to the photocatalytic activity of the ZnO nanoparticles, which degrade the MB dye when exposed to sunlight. Before Treatment (Fig. 7a) in this initial spectrum, the high absorbance peak at around 660–680 nm represents the unaltered, concentrated MB dye. This peak intensity suggests the presence of the dye at a high concentration in the solution. The peak around 300 nm may also be visible due to conjugated π-electrons in the MB dye structure, which absorb in the UV region. After Treatment (Fig. 7b) after exposure to ZnO nanoparticles under sunlight, the UV–Vis spectrum reveals a marked reduction in absorbance at the characteristic wavelength of MB^40,41^. This decrease in absorbance shows that ZnO nanoparticles have effectively catalyzed the degradation of the dye, lowering its concentration in the solution^41^. The absence or reduction of the characteristic MB peak indicates that the dye molecules are breaking down into smaller, non-chromophoric fragments, which no longer absorb at the same wavelengths^42^. The observed changes in the spectra before and after treatment illustrate the efficiency of ZnO nanoparticles in removing dye from aqueous solutions. ZnO is a well-known photocatalyst that, under sunlight, generates electron–hole pairs. These pairs interact with water molecules to produce highly reactive oxygen species (ROS) like hydroxyl radicals, which attack and decompose the dye molecules^43^. This process, known as photocatalytic degradation, ultimately breaks down the dye into less harmful or colorless compounds, which can be measured by the reduction in absorbance at the characteristic wavelength of MB. Also, the significant drop in absorbance in Fig. 7b confirms that the MB dye has been largely decomposed or removed from the solution. The green-synthesized ZnO nanoparticles prove to be effective and sustainable photocatalysts because they utilize natural resources (in their green synthesis) and sunlight, an abundant energy source, to achieve dye degradation. This demonstrates their potential in environmental applications, particularly in wastewater treatment, where organic dyes and pollutants are common contaminants.

### Photodegradation of methylene blue

The photocatalytic activity of ZnO NPs was evaluated by the degradation of MB aqueous solution under direct sunlight.

#### The influence of pH on sunlight photo‑degradation

**Fig. 8:**
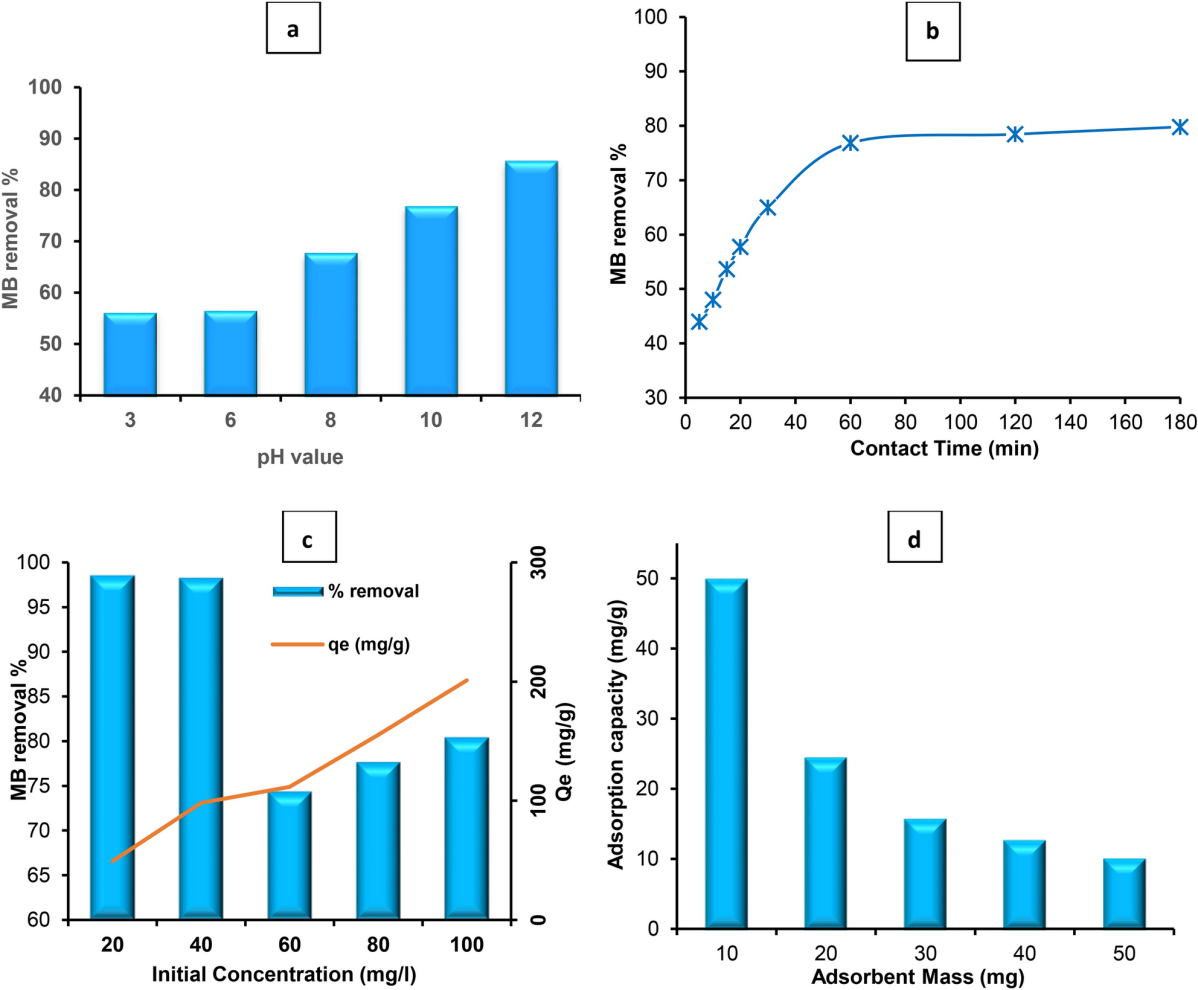
(**a**) The effect of pH, (**b**) Effect of contact, (**c**) Effect of initial concentration of MB and maximum adsorption capacity of ZnO NPs and (**d**) Effect of dosage of green synthetized ZnO NPs on MB photodegradation under direct sunlight photocatalysis.

One of the key elements controlling how well dye is broken down by photocatalysis on the photocatalyst’s surface is pH. It also affects how dye is adsorbed to the photocatalyst’s surface. The provided results in Fig. 8a depict the influence of pH on the removal efficiency of Methylene Blue using ZnO NPs derived from *Padina* algae as a photocatalyst under direct sunlight. The experiments were conducted at pH range of 3–12, and the percentage removal of MB at each pH level was measured. MB degraded photocatalytically at a slow rate at pH 3 and the removal percentage was 55.89%. and at pH 6, the MB removal percentage slightly increased to 56.23%. While, at pH 8, there was a noticeable improvement in MB removal, with a percentage of 67.51%. Correspondingly, at pH 10, the removal efficiency increased significantly to 76.60%. Finally, at pH 12, the MB removal reached its peak efficiency, with a percentage of 85.35%. MB removal increases steadily with increasing pH. Starting from around 56% at pH 3 and reaches a maximum of 85% at pH 12. This tendency is due to the interplay between the surface charges of MB and the ZnO NPs which MB has a pKa (acid dissociation constant) of around 3.5^44^. At lower pH (3–6), the solution is acidic, causing both MB and ZnO NPs to be positively charged. This reduces electrostatic attraction between them, hindering MB adsorption. As pH increases (pH 8–12), the solution becomes more alkaline. While, the ZnO nanoparticle surface becomes increasingly negative. This creates a strong electrostatic attraction between negatively charged ZnO NPs enhancing the adsorption process^45^. Nonetheless, high removal (pH 10–12) which the strong negative charge on ZnO NPs at higher pH facilitates maximum attraction and adsorption of MB molecules.

Correspondingly, low removal (pH 3–6) shows the similar positive charges on both MB and ZnO at lower pH create repulsion, hindering effective adsorption. Furthermore, direct sunlight exposure likely enhances the photocatalytic activity of ZnO NPs, contributing to the degradation of MB molecules adsorbed on the surface of the nanoparticles^46^. This synergistic effect of sunlight and pH on the removal process could explain the higher efficiency observed under direct sunlight conditions compared to previous studies conducted without sunlight exposure. Also, determining the zeta potential of the nanoparticles at various pH values would provide insights into the surface charge behavior and identify the isoelectric point (pH at which the zeta potential is zero).

#### Effect of contact time on photodegradation process

Contact time is a crucial parameter in adsorption processes, as it determines the time required for the adsorbent to effectively remove contaminants from the solution^47^. Optimizing contact time ensures efficient contaminant removal while minimizing process duration and cost. The experiment aimed to investigate the effect of contact time on the removal of Methylene Blue dye by green synthetized ZnO NPs in an aqueous solution under direct sunlight without agitation. The samples were taken at various contact times range 5–180 min, and the percentage removal of MB dye was presented in the Fig. 8b. At longer contact times (60–180 min), the percentage removal of MB dye shows a significant increase, reaching values ranging from 76.835% to 79.798%. This indicates that extended exposure of the MB dye solution to the adsorbent results in enhanced removal of the dye. The increase in removal efficiency at longer contact times can be attributed to the prolonged interaction between the dye molecules and the ZnO NPs surface, allowing more MB dye molecules to be adsorbed and subsequently degraded from the solution^48^. While the low results were showed at shorter contact times (5–30 min), the percentage removal of MB dye is relatively lower, with values ranging from 43.973% to 64.983%. It enabled the dye ions to attach to the adsorbent quickly. Adsorption occurs quickly and is often managed using the diffusion approach from the surface’s bulk solution^49^. When the MB particles are transferred from the external to the internal sites of the ZnO NPs adsorbent molecules, the adsorption increases with an increase in contact time. This is made possible by a larger surface area of the ZnO NPs being available at the beginning and exhaustion of the conversion of external adsorption sites^16^. This suggests that shorter exposure times result in less efficient removal of the dye from the solution. The lower removal efficiency at shorter contact times may be due to insufficient time for the dye molecules to interact with the ZnO NPs surface and undergo adsorption. As a result, fewer dye molecules are removed from the solution within the shorter time frames. Finally, the fast adsorption kinetics and high removal efficiency make this material a promising candidate for wastewater treatment applications^50^.

#### Effect of initial concentration of MB dye on the photodegradation process

The initial concentration of MB dye in aqueous solutions is an important parameter in determining the efficiency of dye removal processes. Understanding the effect of initial concentration allows for the optimization of experimental conditions to achieve maximum removal efficiency and adsorption capacity. The effect of initial MB concentration on the removal efficiency was studied as presented in Fig. 8c, with concentrations ranging from 20 to 100 ppm. The results indicate a notable variation in the percentage removal and equilibrium adsorption capacity (*Qe*) of MB dye as the initial concentration changes. At lower initial concentrations (20 ppm), the removal efficiency was extremely high, reaching 98.556%. This suggests that the photocatalytic process using green synthetized ZnO NPs from *Padina* algae is highly effective in removing MB dye even at low concentrations. The corresponding equilibrium adsorption capacity (*Q*_*e*_) at this concentration is 49.28 mg g^-1^, indicating the amount of MB dye adsorbed per unit mass of the adsorbent. As the initial concentration of MB dye increased, the removal efficiency remained high, with values above 98% even at 40 ppm and 60 ppm. However, at higher concentrations (80 ppm and 100 ppm), the removal efficiency slightly decreased to 77.662% and 80.433%, respectively. Despite this decrease, the process still exhibited significant effectiveness in removing MB dye. The equilibrium adsorption capacities also increased with higher initial concentrations of MB dye, reaching a maximum of 201.08 mg g^-1^ at 100 ppm. This suggests that higher concentrations of MB dye in the solution lead to increased adsorption onto the ZnO NPs surface.

The variation in removal efficiency and equilibrium adsorption capacity with changes in the initial MB concentration can be attributed to several factors^51^. At lower concentrations, there are more active sites available on the surface of the ZnO NPa from *Padina* algae for adsorption and photocatalytic degradation of MB molecules^34^. However, as the initial MB concentration increases, the number of dye molecules in the solution also increases^52^, leading to a higher probability of recombination of photogenerated electron–hole pairs on the surface of the photocatalyst. This can reduce the photocatalytic activity and consequently lower the removal efficiency, as evidenced by the slight decrease observed at higher concentrations. The enhanced adsorption capacity may be the consequence of increased contact between the dye and adsorbent, in addition to an increase in the driving force of the concentration gradient caused by the initial concentration increase^53^. Correspondingly, the increase in equilibrium adsorption capacity with higher initial concentrations can be explained by the Langmuir adsorption isotherm, which suggests that as the concentration of MB dye increases, more dye molecules are adsorbed onto the surface until saturation is reached^54^. This is reflected in the higher *Q*_*e*_ values observed at higher initial concentrations in the experimental data.

## Influence of ZnO NPs dosage on the sunlight photodegradation

The dosage of ZnO NPs is a critical parameter in determining the effectiveness of dye removal processes from aqueous solutions. Understanding how the mass of the adsorbent influences its capacity for removing dye molecules provides valuable insights for optimizing treatment processes^55^. The experiment aimed to explore the influence of the dosage of ZnO NPs on the removal of Methylene Blue dye through a photocatalytic process under direct sunlight. By varying the dosage of the ZnO NPs from 10 to 50 mg for removing the MB dye from an aqueous solution (Fig. 8d). The concentration of MB dye was maintained at 20 ppm at pH 12. The results improve that at lower dosages of the adsorbent (10 mg and 20 mg), the percentage removal of MB dye is notably high, with values of 97% and 99.5% respectively. This suggests that even relatively small amounts of ZnO NPs are highly effective in removing the dye from solution^56^. Correspondingly, the adsorption capacity at these lower dosages is also relatively high, indicating that a substantial amount of MB dye molecules is adsorbed onto the surface of the ZnO nanomaterial. This emphasizes the efficacy of ZnO NPs as a photocatalyst for dye removal, even at low dosages. While, as the dosage of the adsorbent increases beyond 20 mg, there is a gradual decrease in both the percentage removal and adsorption capacity. This decrease becomes more pronounced at higher dosages (40 mg and 50 mg). The declining efficiency at higher dosages could be attributed to several factors, including increased agglomeration of ZnO NPs, which reduces the available surface area for adsorption and photocatalysis^57^. Additionally, at higher dosages, the active sites for dye adsorption and photocatalytic degradation may become saturated, leading to diminished performance^58^. Further increase in the catalyst amount above 50 mg decreased the activity, which could be due to the phenomenon of light scattering and screening effects^59^. Namely, this can be ascribed to the increased aggregation of particles acting as barriers for the light irradiation.

### Sorption isotherm results

**Fig. 9:**
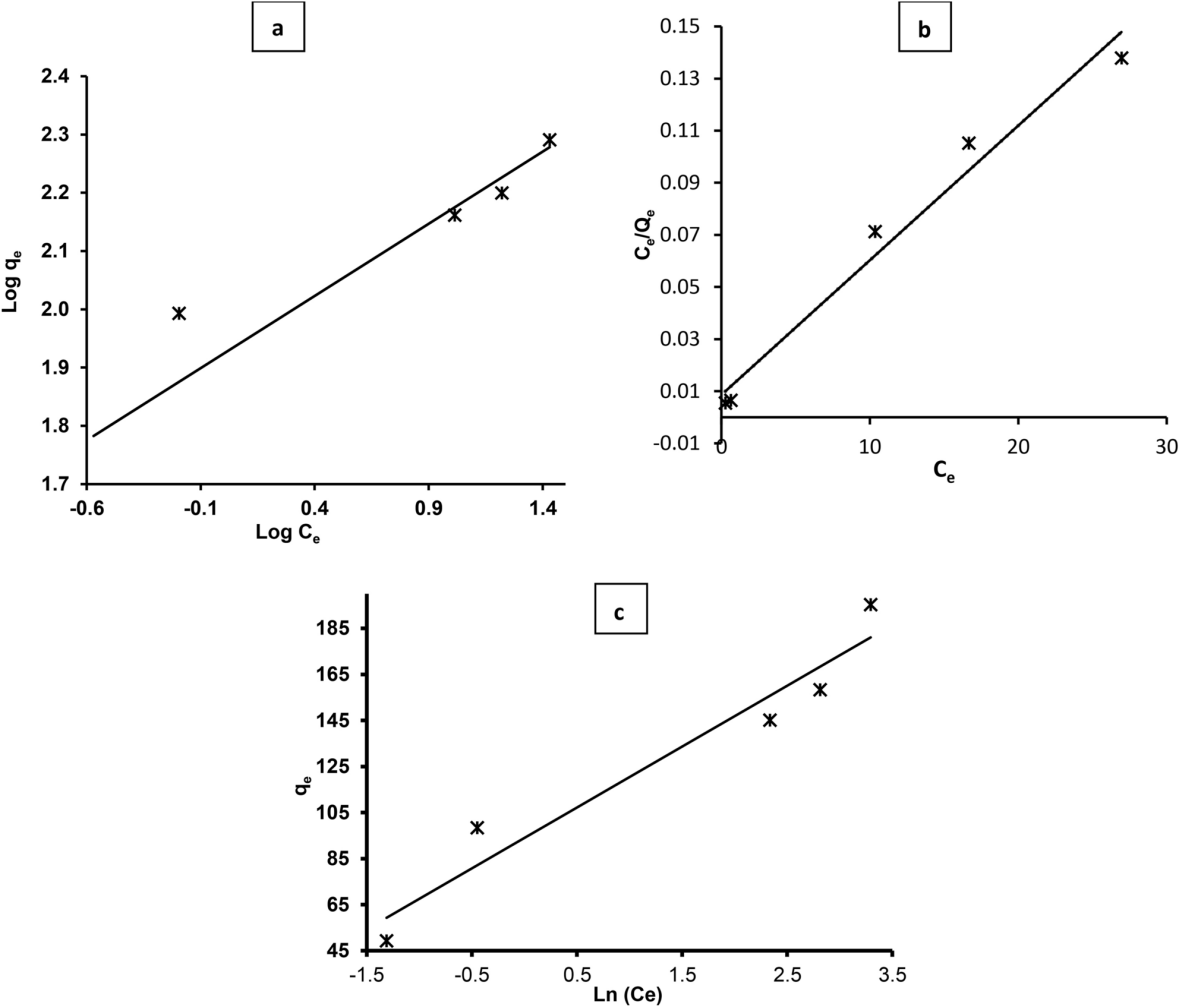
adsorption isotherm models of MB photodegradation on green synthesized ZnO NPs. (**a**) Freundlich isotherm, (**b**) Langmuir isotherm and (**c**) Tempkin isotherm in direct sunlight photocatalysis.

In this study, the sorption equilibrium between the MB dye solution and green synthetized ZnO NPs was investigated using three widely employed isotherm models: Freundlich, Langmuir, and Tempkin. These models were utilized to understand the sorption behavior at different concentrations of the MB dye solution. The data obtained from sorption experiments were fitted to the linear forms of these models, and the resulting constants and correlation coefficients (*R*^*2*^) are presented in the Table 3 and Fig. 9a–c.

**Table 3:** Isotherm constants for MB dye sorption by ZnO NPs.

Isotherm model	Isotherm parameter	Result
Freundlich	*1/n*	0.25
	*K*_*F*_ (mg^1–1/n^L^1/n^g^–1^)	83.91
	*R*^*2*^	0.897
Langmuir	*Q*_*m*_ (mg g^-1^)	192.308
	*K*_*a*_ × 10^3^	604.65
	*R*^*2*^	0.980
Tempkin	*AT*	34.934
	*BT*	26.442
	*R*^*2*^	0.939

#### Freundlich isotherm

The Freundlich isotherm is an empirical model that describes heterogeneous surfaces, where adsorption strength decreases with increasing surface coverage^60^. Table 3 showed the results values of Freundlich constant and parameters. The Freundlich constant (*K*_*F*_) represents the adsorption capacity, while (*1/n*) is a measure of the adsorption intensity. In this study, the value of (*1/n*) is 0.25, indicating a favorable adsorption process since it is less than 1^61^. A lower value of (*1/n*) suggests stronger adsorption at low concentrations. While, the high value of (*K*_*F*_) (83.91 (mg g^–1^)) suggests a high adsorption capacity of the green ZnO NPs for MB dye^62^. Besides, the correlation coefficient (*R*^*2*^ of 0.89) indicates a reasonably good fit of the Freundlich model to the experimental data, although it may not perfectly capture the sorption behavior (Fig. 9a).

#### Langmuir isotherm

The Langmuir isotherm assumes monolayer adsorption onto a homogeneous surface with a finite number of identical sites^63^. )*Q*_*m*_( represents the maximum adsorption capacity of the adsorbent, while )*K*_*a*_( is the Langmuir constant related to the energy of adsorption. The high value of (*Q*_*m*_) 192.308 mg g^-1^ suggests that the ZnO NPs have a high affinity for MB dye molecules, indicating efficient adsorption^34^. In addition, the (*K*_*a*_) value of 604.65 × 10^3^ indicates a strong adsorption affinity between the adsorbate and adsorbent. In addition, the high value of *R*^*2*^ = 0.980 indicates that the Langmuir model provides an excellent fit to the experimental data) Fig. 9b and Table 3), suggesting that the sorption process follows monolayer adsorption behavior.

#### Tempkin isotherm

The Tempkin isotherm accounts for the effects of indirect adsorbate–adsorbate interactions and surface heterogeneity on the adsorption process^60^, (*AT*) represents the Tempkin isotherm constant related to the heat of adsorption, while (*BT*) is related to the adsorption potential. Also, the values of (*AT*) (34.934) and (*BT*) (26.442) as presented in Table (3) indicate moderate adsorption energy and adsorbate-adsorbent interactions. The *R*^*2*^ value of 0.939 in Fig. 9c indicates a good fit of the Tempkin model to the experimental data, suggesting that the model adequately describes the sorption behavior. Finally, according to the *R*^*2*^ values, the Langmuir model provides the best fit for the experimental data, suggesting that monolayer adsorption is the dominant mechanism for MB dye removal by ZnO NPs. This is further supported by the high *Q*_*m*_ value, indicating a significant adsorption capacity. While the Freundlich and Tempkin models also show relatively good fits, they are less accurate than the Langmuir model in describing the adsorption process in this study. In addition, the values of the constants can be affected by various factors, including the properties of the adsorbent and adsorbate, temperature, and pH^64^. For example, a higher *Q*_*m*_ value in the Langmuir model could indicate a higher surface area or more available adsorption sites on the adsorbent. Similarly, a higher *K*_*F*_ value in the Freundlich model suggests a stronger affinity between the adsorbate and ZnO NPs in this study.

### Kinetic models analysis

In this study, five kinetic models were employed to understand the rate of the adsorption process of MB using ZnO NPs. These models include Lagergren first-order, pseudo second-order, intraparticle diffusion, film diffusion, and Elovich models.

#### Lagergren first-order kinetic model

**Fig. 10:**
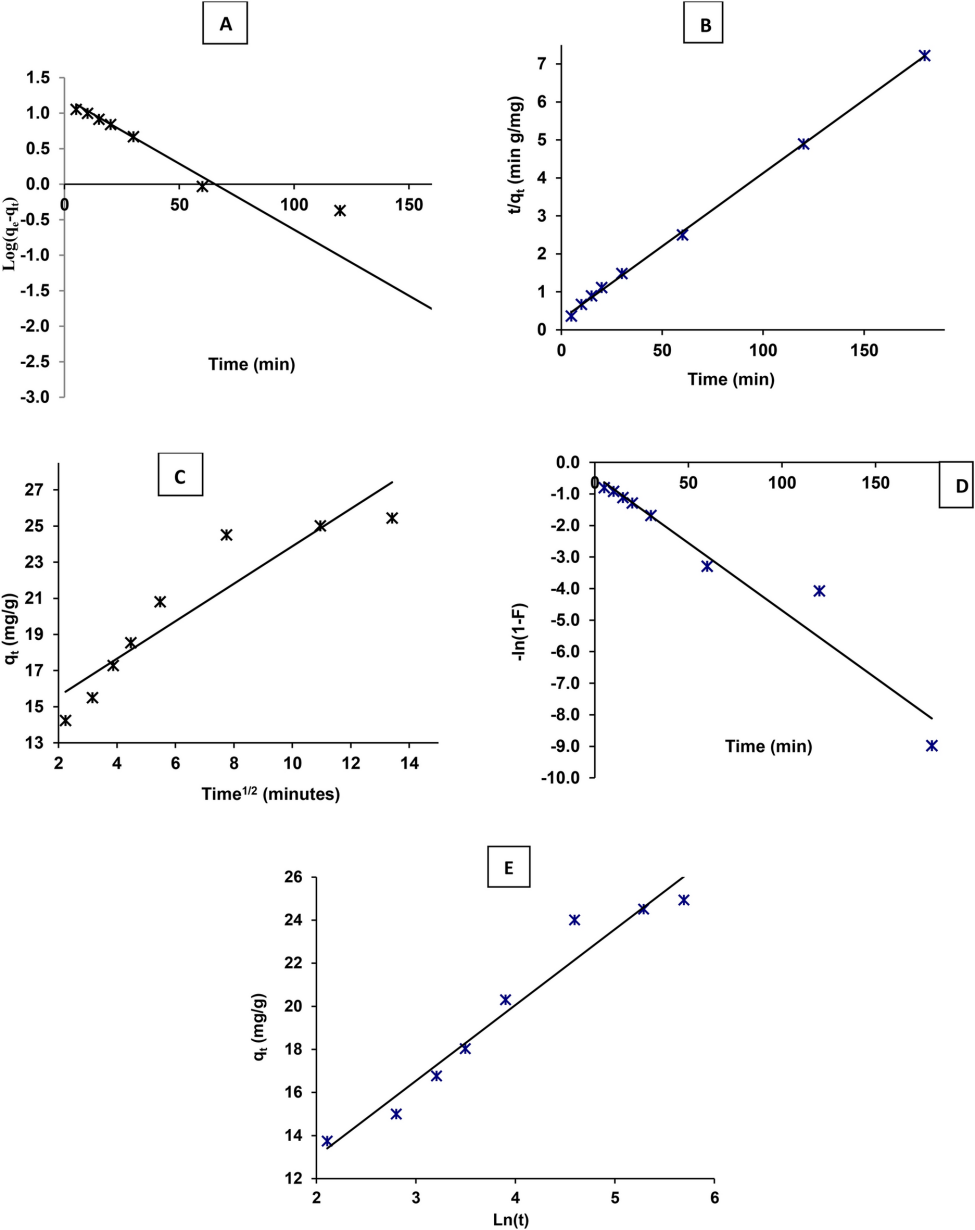
adsorption kinetic models (**A**) Pseudo-first-order, (**B**) Pseudo-second-order, (**C**) intraparticle diffusion, (**D**) Film diffusion and (**E**) Elovich kinetic models of MB photodegradation on green synthesized ZnO NPs. in direct sunlight photocatalysis.

^65^ first-order model helps understand the initial adsorption rate. This model assumes that the adsorption process follows first-order kinetics, where the rate of adsorption is proportional to the number of available adsorption sites^66^. The rate constant (*K*_*1*_) is obtained from the slope of the linear plot of ln (*q*_*e*_—*q*_*t*_) versus time (Fig. 10a), where *q*_*e*_ is the amount of adsorbate adsorbed at equilibrium and *q*_*t*_ is the amount adsorbed at time *t*. In this study, results showed that the (*K*_*1*_) is − 42.84 × 10^3^, indicating a relatively fast initial adsorption rate. However, the calculated equilibrium adsorption capacity (*q*_*e*_) is 16.44, which is lower than expected. In addition, the coefficient of determination (*R*^*2*^) value of 0.943 suggests a good fit of the model to the experimental data, indicating that the first-order kinetics reasonably describe the adsorption process.

#### Pseudo-second-order kinetic model

The pseudo-second-order model suggests that the rate-limiting step of adsorption involves chemisorption, where the adsorbate molecules are held onto the adsorbent surface by chemical bonds^67,68^. Pseudo-second-order model provides insights into chemisorption, which is often the rate-limiting step in adsorption processes^69^. The rate constant (*K*_*2*_) is determined from the slope of the linear plot of *t/q*_*t*_ versus time, where (*t*) is the time and (*q*_*t*_)is the amount of adsorbate adsorbed at time *t* (Fig. 10b). In this study, (*k*_*2*_) is 5.47 × 10^3^, indicating a slower adsorption rate compared to the first-order model. The calculated equilibrium adsorption capacity (*q*_*e*_) using this model is 25.97, which is closer to the experimental values (24.9). In addition, the high coefficient of determination (*R*^*2*^) value of 1.000 as showed in Table (4) which indicates an excellent fit of the model to the experimental data, suggesting that the pseudo-second-order kinetics accurately describe the adsorption process.

#### Intraparticle diffusion model

The intraparticle diffusion model is used to analyze the diffusion of adsorbate molecules within the porous structure of the adsorbent in addition to analyze the mass transfer mechanisms involved in adsorption^70^. The rate constant (*K*_*dif*_) represents the rate of intraparticle diffusion, and (C) is a constant related to the thickness of the boundary layer. In this study, the parameters in Table 4 showed (*K*_*dif*_) is 1.0361, indicating the rate of intraparticle diffusion. However, the coefficient of determination (*R*^*2*^) value of 0.8572 (Fig. 10C) suggests that the model may not fully describe the adsorption process, indicating the presence of additional mechanisms^71^.

**Table 4:** Kinetic parameters for the adsorption of MB dye onto ZnO NPs using different kinetic models.

Kinetic model	Parameters	Value
First-order	*q*_*e*_ (calc.)	16.44
	*q*_*e*_ (Exp.)	24.936
	*k*_*1*_ × 10^3^	− 42.84
	*R*^*2*^	0.943
Second-order	*q*_*e*_ (calc.)	25.97
	*q*_*e*_ (Exp.)	24.936
	*k*_*2*_ × 10^3^	5.47
	*R*^*2*^	1.000
Interaparticle diffusion	*K*_*dif*_	1.0361
	C	13.017
	*R*^*2*^	0.8572
Film diffusion	*K*_*FD*_	0.0428
	*R*^*2*^	0.9433
Elovich	β	0.284
	α	31.723
	R^2^	0.956

#### Film diffusion model

The film diffusion model describes the mass transfer of adsorbate molecules from the bulk solution to the surface of the adsorbent particles through a boundary layer.

The rate constant (*K*_*FD*_) and coefficient of determination (*R*^*2*^) were presented in Fig. 10d and Table 4. In this study, (*K*_*FD*_) is 0.0428, indicating that the specific case signifies that the film diffusion process is moderately fast for the given system. A higher value would indicate that the adsorbate molecules are transferred even quicker from the bulk solution to the surface of the adsorbent particles^72,73^. The coefficient of determination *(R*^*2*^*)* value of 0.9433 suggests a reasonable fit of the model to the experimental data, indicating that film diffusion plays a role in the adsorption process.

#### Elovich kinetic model

The Elovich model considers the adsorption process as a combination of chemisorption and diffusion-controlled processes^67^. α constant represents the initial adsorption rate. It signifies the amount of dye adsorbed per unit area of the adsorbent at zero time. A higher value of α indicates a faster initial adsorption rate. As represented in Fig. 10e and Table 4, in this study, α = 0.284 suggests a moderate initial rate of dye uptake. While, β constant is related to the desorption energy. It reflects the activation energy required for the adsorbed dye molecules to desorb from the surface. A higher β value corresponds to a stronger attraction between the dye and the adsorbent, making desorption less favorable. The results showed that β = 31.723 indicates a relatively high desorption energy, suggesting that once the dye molecules are adsorbed, they are less likely to desorb back into the solution^74^. In this study, the *(R*^*2*^*)* value of 0.956 suggests a good fit of the Elovich model to the experimental data, providing insights into the initial adsorption rate and desorption energy^75^.

Finally, amongst the kinetic models, the pseudo-second-order model provides the best fit to the experimental data, as indicated by the highest coefficient of determination *(R*^*2*^*)* value of 1.0. This suggests that the rate-limiting step of adsorption involves chemisorption, which is consistent with the experimental observations. The pseudo-second-order kinetic model is considered the most suitable for describing the adsorption process, as it accurately represents the experimental data and provides insights into the adsorption mechanism.

## Effect of different light source and photocatalytic activity

**Fig. 11:**
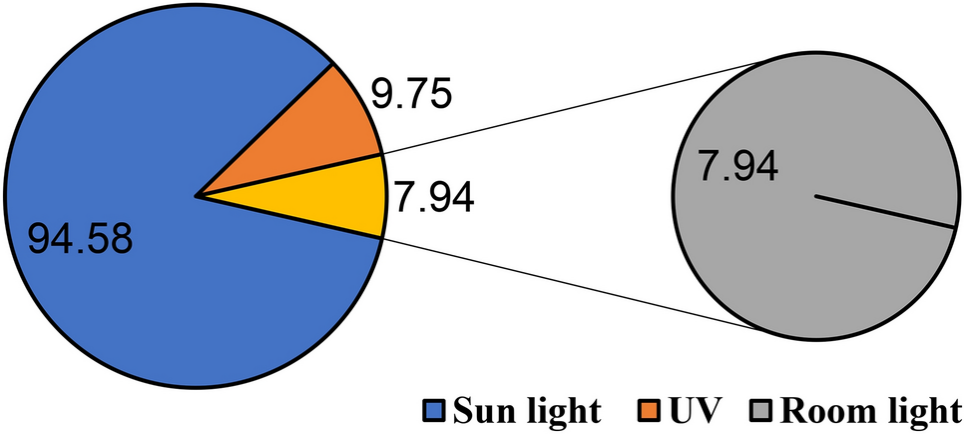
Removal percentage of effect of ZnO NPs in degrading MB under various light conditions.

The type and intensity of light source, pH of the solution, and the presence of other substances can also influence the degradation process. Exploring these factors will provide a more comprehensive understanding of the photocatalytic system. The experiment investigated the effectiveness of green synthesized ZnO nanoparticles in degrading methylene blue under various light conditions (Fig. 11). The results clearly demonstrate that the degradation process is significantly influenced by the type of radiation and the concentration of ZnO NPs.

Sunlight exhibits the highest degradation efficiency (94.58%). This suggests that the photocatalytic activity of ZnO NPs is primarily driven by the UV component present in sunlight. Sunlight’s superiority is primarily due to its broad spectrum, including UV and visible light, which effectively activates the ZnO NPs and generates abundant ROS for MB degradation^76^. The UV component excites electrons from the valence band to the conduction band, creating electron–hole pairs. These pairs participate in redox reactions, leading to the formation of hydroxyl radicals and other ROS, which are the primary agents for breaking down MB molecules^77^.

Negligible degradation occurs in the dark (0.72%) this confirms that the process is primarily photocatalytic, relying on light energy to activate the ZnO NPs. UV light alone results in moderate degradation (9.75%). This indicates that while UV light is crucial, other components in sunlight might also contribute to the enhanced degradation observed. Besides, room light shows minimal degradation (7.94%). which suggests that the intensity or specific wavelengths present in room light are not sufficient light energy for activating the photocatalytic process. Visible light also contributes to the degradation process, albeit to a lesser extent, by exciting electrons to a higher energy state within the conduction band, potentially facilitating additional ROS generation.

ZnO is a well-known semiconductor photocatalyst. Upon absorbing light energy greater than its bandgap energy, it generates electron–hole pairs. These charge carriers participate in redox reactions with adsorbed molecules like MB, leading to their degradation.

The high efficiency under sunlight can be attributed to the broad spectrum of light, including UV and visible regions, which excites a larger number of electrons in ZnO NPs, leading to increased generation of reactive oxygen species like hydroxyl radicals. These ROS are responsible for the oxidative degradation of MB^78^. The minimal degradation under dark and room light conditions confirms the photocatalytic nature of the process. Without sufficient light energy, the generation of electron–hole pairs and subsequent ROS formation is limited, resulting in negligible degradation. Longer contact time between MB and ZnO NPs allows for more interactions and increases the probability of degradation. However, the rate of degradation is not necessarily constant and may slow down over time as the readily available dye molecules are consumed, and the remaining ones become less accessible or require more complex reaction pathways. However, an optimal concentration of ZnO NPs and MB dye exists beyond which the efficiency may decrease due to factors like light scattering, nanoparticle aggregation, and competition for light absorption among the particles^13^. so, the factors influencing the rate of degradation over time include the initial dye concentration, light intensity, and the efficiency of mass transfer within the system.

## Dye MB removal mechanism

**Fig. 12:**
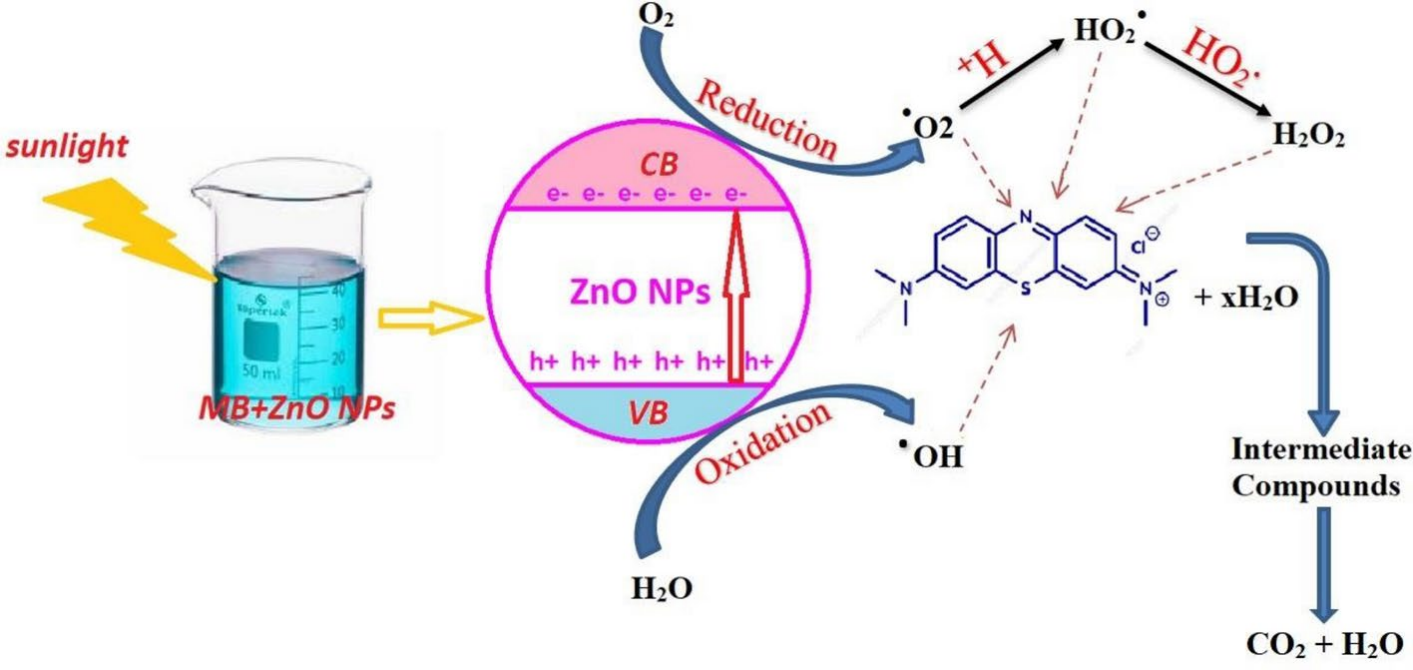
Schematic diagram of photodegradation of methylene blue in ZnO NPs under direct sunlight.

The mechanism of MB dye removal by green ZnO from *Padina* algae as illustrated in Fig. 12, involves physical adsorption and chemisorption processes. Initially, the dye molecules are attracted to the surface of the ZnO material through physical interactions such as electrostatic forces and Van der Waals interactions ^10^.

The extract from *Padina pavonica* brown algae has a significant impact on the properties of the synthesized zinc oxide (ZnO) nanoparticles, ultimately influencing their efficiency as a photocatalyst for methylene blue (MB) dye removal. This effect stems from the presence of bioactive compounds within the algae, such as polysaccharides, polyphenols, and flavonoids, which play multiple roles during synthesis.

The bioactive compounds act as reducing agents, facilitating the conversion of zinc ions (Zn^2+^) from zinc nitrate hexahydrate to metallic zinc (Zn^0^) and subsequently to ZnO nanoparticles. These compounds also act as stabilizing and capping agents, preventing the newly formed ZnO nanoparticles from aggregating. This helps to maintain a higher surface area for adsorption and enhances the stability of the nanoparticles in the solution^79^. The extract contributes to the unique surface properties of the ZnO nanoparticles. This is confirmed by EDX analysis which showed significant carbon content suggesting organic molecules are bound to the surface. This surface modification could lead to improved interaction with the dye molecules due to changes in surface charge and hydrophilicity. Furthermore, the effect of the extract on the properties of ZnO can also be understood by considering these additional benefits: the *Padina pavonica* extract prevents the agglomeration of ZnO nanoparticles, allowing them to maintain smaller sizes, leading to increased surface area. A larger surface area leads to more contact points between the dye molecules and the catalyst, thereby increasing the adsorption efficiency. Also, the bioactive compounds in the algae extract are hypothesized to influence the electronic band structure of ZnO nanoparticles, decreasing the recombination rate of photogenerated electron–hole pairs. This contributes to the observed enhancement of photocatalytic activity under direct sunlight^80^. The extract might also promote improved light absorption by ZnO, as these organic molecules could potentially act as sensitizers for visible light, expanding the photocatalytic process beyond just the UV portion of the spectrum.

Subsequently, chemical bonds may form between the dye molecules and the surface functional groups of the ZnO NPs, leading to stable adsorption^44^. Also, the photocatalytic removal of MB dye using green ZnO from *Padina* algae involves the generation of electron–hole pairs upon exposure to light^76^. These photoexcited charge carriers participate in redox reactions with the dye molecules adsorbed on the surface of the photocatalyst, leading to their degradation into harmless products such as water and carbon dioxide^54^. Additionally, the adsorption of dye molecules onto the surface of the catalyst facilitates their subsequent degradation by providing close proximity to the active sites. The photocatalytic removal of MB dye by ZnO NPs under sunlight involves the generation of electron–hole pairs upon exposure to light. These photoexcited charge carriers participate in redox reactions with the dye molecules adsorbed on the surface of the ZnO, leading to their degradation into harmless byproducts^46^.

However, the SEM analysis reveals ZnO NPs with an average size ranging from 16.34 to 22.88 nm. Smaller particle size is often associated with increased surface area and enhanced photocatalytic activity. Therefore, the observed photocatalytic efficiency might be attributed to the relatively small particle size and a high surface area.

The primarily spherical morphology observed in SEM suggests potential for increased surface area compared to other morphologies. This can lead to more effective contact points with the MB dye and potentially higher efficiency of ROS generation.

The presence of significant carbon content observed in the EDX analysis might be a consequence of organic molecules present on the surface of ZnO NPs. While this study did not investigate the specific types of organic molecules present, it’s worth considering that these molecules can impact surface charge and hydrophilicity, potentially playing a role in adsorption and interaction with MB dye. The specific nature of these surface-bound organic compounds could be investigated in future studies.

Zinc Oxide is a wide-bandgap semiconductor with a bandgap energy of approximately 3.37 eV. Under direct sunlight, which contains a significant amount of ultraviolet (UV) radiation, ZnO nanoparticles can absorb photons that have energy equal to or greater than the bandgap energy.

$$ZnO + h\nu \to e^{ - } + h^{ + }$$

This equation represents the generation of an electron–hole pair when the ZnO nanoparticle absorbs a photon of light. The photoexcitation process results in the formation of an electron (e^−^) in the conduction band and a hole (h^+^) in the valence band.

The photoexcited electron and hole participate in subsequent redox reactions that generate various reactive oxygen species (ROS). These ROS, particularly hydroxyl radicals (^∙^OH) and superoxide radicals (O^2^), are highly reactive and play a critical role in the degradation of Methylene Blue.

The photoexcited electron reduces molecular oxygen (O_2_) present in the aqueous medium to form superoxide radicals. The holes in the valence band can react with hydroxyl ions (OH^−^) or water molecules (H_2_O) to generate hydroxyl radicals.

$$H_{2} O + h^{ + } \to OH + H^{ + }$$

$$OH^{ - } + h^{ + } \to OH$$

The generated ROS, particularly hydroxyl and superoxide radicals, have a high oxidative potential and are capable of attacking the Methylene Blue (MB) dye molecules, leading to their degradation. The degradation involves breaking down the conjugated chromophore system of MB, which is responsible for its intense blue color. Hydroxyl radicals are strong oxidants that attack the aromatic rings in MB, leading to the cleavage of the bonds and ultimately breaking down the dye into smaller, less harmful molecules.

$$MB + OH \to Degradation \Pr oducts$$

Superoxide radicals can also participate in the oxidative degradation of MB, especially through indirect pathways, by forming hydrogen peroxide (H^2^O^2^), which further contributes to the formation of more hydroxyl radicals.

$$2HO_{2} \to H_{2} O_{2} + O_{2}$$

$$H_{2} O_{2} + h\nu \to 2OH$$

To better understand the role of ROS in the photocatalytic degradation process, scavenging experiments can be conducted. In such experiments, specific scavengers (such as tertiary butanol for hydroxyl radicals or p-benzoquinone for superoxide radicals) are introduced into the system to selectively inhibit the activity of these species. The decrease in photocatalytic efficiency when these scavengers are added confirms the involvement of ROS in the degradation of MB.

For instance:

It can quench hydroxyl radicals, leading to a marked decrease in the degradation rate of MB, indicating that ∙OH is a key player in the photocatalytic process.

$$OH \, + \, Scavenger \, \to \, Inactive Species$$

Also, scavengers play a crucial role in photocatalytic degradation experiments as they help to identify the reactive species (radicals) responsible for the degradation process. They are specific compounds that react with and effectively “deactivate” certain types of radicals. By observing how the addition of scavengers impacts the degradation rate, researchers can deduce the contribution of those radicals to the overall degradation process^81^.

Hydroxyl Radicals (∙OH), these are considered highly reactive and play a major role in the oxidation of organic compounds, including MB. The text describes hydroxyl radicals forming through reactions involving photogenerated holes with either hydroxyl ions (OH^−^) or water molecules.

Superoxide Radicals (O_2−_), while less directly involved in MB oxidation, they can contribute to degradation indirectly. Superoxide radicals react to form hydrogen peroxide (H_2_O_2_), which then decomposes under light conditions to generate more hydroxyl radicals. For instance, the authors of the study used tertiary butanol to act as a scavenger for hydroxyl radicals and observed a decreased degradation rate, confirming ∙OH involvement in the degradation process. Another commonly used scavenger is p-benzoquinone, which traps superoxide radicals.

## Antibacterial activity of green synthesized ZnO NPs

The emergence of antibiotic-resistant bacteria poses a significant threat to public health. Waterborne pathogens are a particular concern, as contaminated water can spread infections. This research explores the potential of green synthesized ZnO NPs derived from *Padina* brown algae as an antibacterial agent for water treatment, offering a sustainable and ecofriendly alternative to conventional methods. Green synthesized ZnO NPs could provide an effective and environmentally friendly approach to disinfection. The results of a laboratory study, summarized in the Table 5, indicate the sensitivity levels of each species to the ZnO NPs.

**Table 5:** Antibacterial activity of green synthesized ZnO NPs from *Padina* brown algae against various bacterial species.

Bacterial species	Sensitivity
*Staphylococcus aureus* ATcc25923	Resistant
*Escherichia coli* ATcc8731	Highly sensitive
*Pseudomonas aeruginosa* ATcc9027	Highly sensitive
*Vibrio damsella*	Highly sensitive
*Klebsiella pneumoniae* ATcc13883	Highly sensitive
*Enterococcus faecalis* ATcc29212	Intermediate

The findings highlight the potential of green synthesized ZnO NPs from *Padina* algae for water treatment applications: which the observed antibacterial activity against a diverse range of bacterial species, including both Gram-positive and Gram-negative bacteria, indicates a potential for broad-spectrum water disinfection. This observation can be attributed to the distinct biochemical composition of the cell walls of the different bacterial species. The high sensitivity of *Escherichia coli, Pseudomonas aeruginosa, Vibrio damsella, Klebsiella pneumoniae,* and *Streptococcus agalactiae*, which are often found in contaminated water, suggests that ZnO NPs could effectively reduce the risk of waterborne infections. Also, the effectiveness against *Staphylococcus aureus*, a bacterium often exhibiting antibiotic resistance, emphasizes the potential of ZnO NPs in addressing this growing public health issue. The cytoplasmic membrane of Gram-positive bacteria is formed of an internal phospholipid layer, which is followed by a thick coating of peptidoglycan. ZnO NPs penetration may be hindered by these thick peptidoglycan coatings^82^.

The antibacterial activity of ZnO NPs is likely attributed to multiple mechanisms: ZnO NPs can generate ROS, which are highly reactive molecules that can damage bacterial cell membranes and lead to cell death. Because ROS have oxidizing qualities that can harm bacterial biological components like amino acids, DNA, proteins, lipids, and ribosomes, they have an inhibiting effect^83^. Correspondingly, ZnO NPs can disrupt the bacterial cell wall, leading to leakage of intracellular components and cell lysis. Likewise, ZnO NPs can potentially penetrate bacterial cells and cause DNA damage, inhibiting bacterial replication. As a result of the direct interaction between ZnO–NPs and bacterial cells, the cytoplasmic membrane’s selective permeability may be altered by changing the channels and transporter proteins that overlap with the bacterial cell membrane^84^. When ZnO-NPs dissolve inside the bacterial cell, toxic ions like Zn^2+^ are produced. These ions can negatively impact enzyme active sites involved in metabolism, amino acid synthesis, and/or proton motive force. In the end, the active transit system may be stopped. Any one of these dysfunctions can lead to the death of bacterial cells^85^. These mechanisms suggest that ZnO NPs could effectively disinfect water by ZnO NPs can directly interact with and kill bacteria in contaminated water. ZnO NPs can potentially remain active in water for extended periods, providing long-lasting disinfection effects. Their use aligns with sustainable water management principles, minimizing the use of harmful chemicals.

## Comparative study of green ZnO nanocomposites for MB dye removal

Based on the data presented in Table 6, the current study the observed high adsorption capacity (Qm = 192.308 mg g^−1^) and excellent removal efficiency (> 98%) for MB at low dye concentrations are attributed to the successful green synthesis of ZnO NPs using the extract of Padina pavonica brown algae. This signifies the potential of this specific nanocomposite for efficient dye removal applications. Maintaining a removal efficiency above 98% even at higher dye concentrations (40 ppm and 60 ppm) further strengthens its effectiveness. The algae are rich in bioactive compounds such as polysaccharides, polyphenols, and flavonoids. These compounds played a significant role in the synthesis process, acting as reducing and stabilizing agents, as well as influencing the final properties of the ZnO NPs. Specifically, the presence of these bioactive compounds likely contributed to: the bioactive compounds, acting as capping agents, may have prevented excessive agglomeration of the ZnO nanoparticles. This resulted in a higher surface area, enhancing the adsorption capacity of the ZnO NPs for MB dye molecules. Bioactive compounds in the algae extract could potentially influence the surface properties and electronic band structure of the ZnO nanoparticles. This may lead to a decrease in the recombination rate of photogenerated electron–hole pairs, which is a major factor limiting the efficiency of photocatalytic processes. This effect likely contributed to the enhanced photocatalytic activity observed in this study.

**Table 6:** Comparative Table of zinc oxide based for removal of dyes by photocatalysis process.

The study	Adsorbent (MONPs)	Algal species	Organic pollutant	Q_m_ (mg/g) or removal %	Optimization condition	Reference
Current Study	ZnO	Padina sp.	Methylene Blue (MB)	Qm (192) & 98%	40 ppm and 60 ppm	Current Study
Rajaboopathi & Thambidurai, 2017	CdO-ZnO	*Padina gymnospora*	Reactive Blue 198	99.57%	15 min	^86^
Sharma et al., 2018	TiO2	*Chlorella pyrenoidosa*	Crystal Violet	–	150 min	^87^
Rabie et al., 2020	CO-ZnO	*Sargassum species*	Malachite Green	–	10 min; 0.05 g; pH 7	^88^
Ishwarya et al., 2018	Zn–O	*Ulva lactuca*	Methylene Blue	90%	120 min	^89^
Subramanian et al., 2022	ZnO	*Sargassum muticum*	Methylene Blue	96%	60 min	^90^
Khalafi et al., 2019	ZnO	*Chlorella species*	Dibenzothiophene	97%	3 h; Room temp; pH 7	^91^
Fouda et al., 2022b	ZnO-NPs	*Ulva fasciata*	Methylene Blue	84.9%	140 min; 35 °C; pH 7	^92^

Compared to other metal oxides like TiO_2_ and MgO, the ZnO from the current study demonstrates superior performance in terms of adsorption capacity and removal efficiency for MB. Also, the choice of algal species for green synthesis seems to influence the adsorption performance. ZnO derived from Padina sp. shows better results than those from *Ulva lactuca* and *Sargassum muticum* for MB removal. While, the doping ZnO with cobalt (Co–ZnO) appears to improve the removal efficiency for Malachite Green, achieving similar results in a shorter time compared to undoped Nano-ZnO.

Founded on the contact time parameter in Table 6, most studies show a positive correlation between longer contact times and higher removal efficiency (consistent with the idea that more time allows for greater interaction), a few studies illustrate exceptions. For example, Rabie et al.^88^ and Subramanian et al.^90^ achieve high removal percentages (99.57% and 96% respectively) in relatively short durations (10 min and 60 min). This suggests that these specific materials and dye combinations reach equilibrium faster, perhaps due to factors like higher surface area or a stronger adsorption affinity. Khalafi et al.^91^ employ the longest contact time (3 h), potentially impacting their efficiency (97% removal) but emphasizing that equilibrium is possible with varying kinetics. Overall, while the general trend suggests greater removal with longer time, some variations exist, possibly influenced by specific materials or experimental setup.

Also, a clear pattern for initial dye concentration and removal efficiency isn’t consistently observed. While the current study showcases slightly decreasing removal efficiency at higher dye concentrations (80–100 ppm) indicating saturation effects, many other studies don’t provide detailed concentration-dependent data. This lack of consistent trends likely results from the diversity of adsorbents and dyes, necessitating a case-by-case analysis of each system’s specific behavior.

While, for pH parameter, the current study provides a strong demonstration of the pH effect, revealing increased removal at higher pH values, typical for ZnO’s adsorption behavior with cationic dyes. However, other studies often don’t focus on pH effects or explore its impact. Adsorbent Dosage: It’s less common for studies to systematically vary adsorbent dosage to understand the effect on removal. While the current study suggests a reduction in efficiency after a certain dosage threshold, a comprehensive comparison would need similar experimental designs across various studies for a reliable analysis.

Also, the *Padina pavonica*-derived ZnO NPs show a significantly higher Qm (192.308 mg/g) for MB than any other reported ZnO or ZnO composite. While Rajaboopathi & Thambidurai^86^ report excellent removal (99.57%), they don’t provide Qm data. However, comparing this high value suggests that *Padina pavonica*-based ZnO possesses a strong ability to adsorb dye molecules, potentially contributing to its superior overall performance.

Although several other studies in Table 6 report near-complete removal efficiencies, like Ishwarya et al.^89^ at 90% and Subramanian et al. (2022) at 96%, these studies highlight similar levels of performance but in varying timeframes and conditions. While Khalafi et al.^91^ report a slightly lower efficiency (97%) for Dibenzothiophene, it’s essential to acknowledge that they are targeting a different pollutant. Comparing the *Padina pavonica*-derived ZnO to other systems (particularly ZnO-based composites), it exhibits noteworthy overall performance for MB dye removal, potentially due to a combination of high adsorption capacity, a “green” synthesis method using algae, and possible synergistic effects from the bioactive compounds.

## Conclusion

This research successfully demonstrated the synthesis of highly efficient and environmentally friendly zinc oxide nanoparticles (ZnO NPs) using *Padina pavonica* brown algae extract. These green-synthesized ZnO NPs exhibited remarkable adsorption capacity (Qm = 192.308 mg/g) and achieved a removal efficiency exceeding 98% for methylene blue (MB) dye, particularly at low dye concentrations (20–60 ppm). These results showcase the potential for wastewater treatment applications. The Langmuir isotherm model provided the best fit for the adsorption process, suggesting monolayer adsorption, while the pseudo-second-order kinetic model accurately described the rate-limiting step, highlighting chemisorption as the primary mechanism. Furthermore, ZnO NPs displayed superior photocatalytic activity under direct sunlight (94.58% removal efficiency) compared to other light sources (UV, and room light conditions). This superiority is likely attributed to the broad spectrum of sunlight, which efficiently excites electrons in ZnO NPs, generating abundant reactive oxygen species (ROS) for MB degradation. Additionally, the green synthesized ZnO NPs demonstrated broad-spectrum antibacterial activity against both Gram-positive and Gram-negative bacteria (e.g., *Escherichia coli*, *Pseudomonas aeruginosa*, *Vibrio damsella*, *Klebsiella pneumoniae*, and *Enterococcus faecalis*). Notably, high sensitivity was observed against common water contaminants, suggesting a potential for effective risk reduction of waterborne infections. Furthermore, these NPs exhibited a potential for addressing antibiotic-resistant bacteria like *Staphylococcus aureus*. The study offers a sustainable and eco-friendly approach for wastewater treatment. The green synthesized ZnO NPs demonstrate exceptional adsorption and photocatalytic capabilities along with potential for antibacterial applications, making them a promising solution for various environmental remediation challenges.

## Data Availability

All data during this study are included in this article and this manuscript does not report data generation or analysis.
